# Comparison of the oxidative stability of soybean and sunflower oils enriched with herbal plant extracts

**DOI:** 10.1007/s11696-018-0516-5

**Published:** 2018-05-28

**Authors:** Mariola Kozłowska, Eliza Gruczyńska

**Affiliations:** Faculty of Food Sciences, Department of Chemistry, Warsaw University of Life Sciences (WULS-SGGW), Nowoursynowska 159C St, 02-776 Warsaw, Poland

**Keywords:** Sunflower oil, Soybean oil, Herbal plant extracts, Oxidative stability, Differential scanning calorimetry

## Abstract

The present study was conducted to determine and compare the oxidative stability of soybean and sunflower oils using differential scanning calorimetry (DSC). These edible oils were enriched with marjoram (*Origanum majorana* L.), thyme (*Thymus vulgaris* L.), and oregano (*Origanum vulgare* L.) extracts at three different concentrations and synthetic antioxidant (BHA). The fatty acid composition of studied oils was determined by gas chromatography mass spectrometry to evaluate the content of unsaturated fatty acids that are sensitive to oxidation process. Oil samples were heated in the DSC at different heating rates (4.0, 7.5, 10.0, 12.5, and 15.0 °C min^−1^) and oxidation kinetic parameters (activation energy, pre-exponential factor, and oxidation rate constant) were calculated. The results showed that the oxidative stability of sunflower oil samples enriched with oregano extracts and soybean oil supplemented with thyme extracts was improved compared to samples without the addition of herbal plant extracts and the synthetic antioxidant.

## Introduction

Sunflower and soybean oils belong to the popular vegetable oils used in the food, cosmetic, and pharmaceutical industries (Rabasco Alvarez and González Rodríguez 2000). They are a good source of the essential fatty acids and liposoluble vitamins which are important components in human diet. The predominant fatty acids present in both oils are unsaturated ones such as oleic, linoleic, and linolenic acids. The presence of double bound in these fatty acids can offer several possibilities to carrying out of the chemical modification of the structure to improve some of their properties (Naeli et al. 2017). On the other hand, they are prone to oxidative processes both when vegetable oils are stored at low temperature as well as when they are used in the kitchen for frying and cooking at high temperature. Autoxidation of edible oils and fats can also be catalyzed by other factors such as exposure to light, heat, and transitional metals. This process is a free radical chain reaction, leading to increase in reactive radicals, which initiate further reactions (Choe and Min 2006; Taghvaei and Jafari 2015). They cause a sequence of unfavorable changes, mainly deterioration in the sensory properties, decrease in nutritional value, and give rise to chemical compounds that are harmful to the human health (McClements and Decker 2000; Gramza-Michalowska et al. 2007).

The lipid oxidation may be inhibited by different ways including inactivation of enzymes catalyzing oxidation, addition of chelating agents or the use of suitable packaging. Another method is the addition of antioxidants, especially natural, because the prolonged usage of synthetic antioxidants has often been questioned due to potential toxicological concerns (Nieva-Echevarría et al. 2015). Antioxidants added to edible oils should protect unsaturated fatty acids to increase their stability to thermal degradation as well as they should demonstrate good thermal stability. Extracts from herbal plants such as thyme, rosemary, sage, marjoram, and oregano are a rich source of natural antioxidants (Oliveira et al. 2018; Chrpová et al. 2010; Generalić Mekinić et al. 2014). Their properties are determined by the presence of phenolic compounds which extend the shelf-life of food, protect fats against autoxidation, and also exhibit antimicrobial activity (Kozlowska et al. 2015). Phenolic compounds may act as antioxidants by scavenging of free radicals. They are active when are used at an optimal range of concentrations. The use of their in excessive amounts may result in pro-oxidant effect. Moreover, herbal plant extracts contain many compounds that may interfere with one another and minor lipid components and also can influence on the autoxidation process.

To determine of the oxidative stability of oils, as well as the antioxidant effectiveness of spices and herbs many methods and techniques were adopted. The most popular methods are peroxide value and conjugated diene determinations, gas chromatography, chemiluminescence, Raman spectroscopy, or Rancimat test (Farhoosh 2007; Carmona et al. 2014). Among them, also differential scanning calorimetry (DSC) is the most used analytical instrument for studies of physical properties and thermo-oxidative decomposition of native and inhibited fats and their blends (Thurgood et al. 2007; Kozłowska et al. 2014; Tengku-Rozaina and Birch 2016). Both isothermal (constant temperature) and non-isothermal (linear increase in temperature) DSC techniques may be applied to obtain kinetic parameters of lipid oxidation in vegetable oils including activation energy (*E*_a_), the Arrhenius rate constant (*k*), pre-exponential factor (*Z*), and also induction time (*τ*). They are calculated using the Ozawa–Flynn–Wall method (OFW) after determination from DSC curves of the onset temperatures (*t*_ON_) which are taken as a parameter characterizing the oxidative susceptibility of oils. Spice and herbs added to vegetable oils act as antioxidants mainly by increasing the onset temperatures. The correct use of the OFW method in DSC studies is based on the comparison of systems at the same degree of conversion (*α*) described as *α* = Δ*H*_τ_/Δ*H*_total_, where Δ*H*_τ_ is the heat evolved at a specific time *τ* and Δ*H*_total_ is the heat evolved during the process. It may be assumed that the degree of conversion at the beginning of the oxidation process is low but constant (Guimarães-Inácio et al. 2018). The approach proposed by Ozawa, Flynn, and Wall is often used during the evaluation of the stability of edible oils and researchers agree that the determination of a single activation energy at the start of the oil degradation is sufficient to compare different samples that were subjected to the same experimental conditions (same heating rates, oxidizing atmosphere) (Thurgood et al. 2007; Guimarães-Inácio et al. 2018).

The aim of this study was to evaluate and compare the oxidative stability of two vegetable oils with and without the addition of herbal plant extracts by DSC non-isothermal measurements. The kinetic parameters were calculated and they also used for evaluation of plant antioxidant efficiency.

## Experimental

### Materials

Refined sunflower (SFO) and soybean (SBO) oils were bought from a local market. The dried leaves of marjoram (*Origanum majorana* L.), thyme (*Thymus vulgaris* L.), and oregano (*Origanum vulgare* L.) were purchased from a local food store in Warsaw. All the solvents (*n*-hexane, methanol, ethanol, acetone, diethyl ether, and chloroform) and reagents (potassium hydroxide, acetic acid, butylated hydroxyanisole—BHA, and certified fatty acids methyl ester reference standard mixture) were of analytical grade and used without further purification. They were purchased from Avantor Performance Materials (Gliwice, Poland) and from Sigma-Aldrich Chemicals (Poznań, Poland).

### Preparation of extracts

The herbal plant extracts were prepared from marjoram, thyme and oregano according to method described by Kozłowska et al. (2010). 10 g of each dried plant material was mixed with ethanol/water (7:3, v/v) in oil bath for 10 h at 45 °C. Next, the filtration was carried out to separate the plant residue, and then, the solvent was removed to dryness in a rotary evaporator at 40 °C. Obtained herbal plant extracts were stored frozen until further use (− 20 °C).

### Determination of peroxide and acid values

Analyses for peroxide and acid values (PV and AV, respectively) were carried out in triplicate according to the Standards ISO 3960 (2009) and ISO 660 (2010).

### Determination of fatty acid composition

Fatty acid composition was analyzed by gas chromatography (GC) after derivatization to fatty acid methyl esters with 2 M methanolic solution of potassium hydroxide according to ISO 12966-2 (2011). A Shimadzu GC-17A gas chromatograph equipped with a flame ionization detector and a BPX capillary column (30 m × 0.22 mm × 0.25 µm film thickness) was used. The analysis was performed using nitrogen (1 mL min^−1^) as the carrier gas and applying the following temperature programme: 60 °C held for 1 min, after which the temperature was increased to 170 °C at a rate of 10 °C min^−1^ and from 170 to 230 °C at a rate of 3 °C min^−1^. The temperature was kept at 230 °C for another 15 min. The injector and detector temperatures were set at 225 and 250 °C, respectively. Individual fatty acids were identified by comparing their retention times with a certified fatty acids methyl ester reference standard mixture (Supelco 37-Component Fame Mix, CRM47885, Sigma-Aldrich, St. Louis, MO, USA) and quantified as a percentage of the total fatty acids.

### Preparation of oil samples

The herbal plant extracts were added to sunflower and soybean oils as solutions in absolute ethanol in the following quantities: 0.01, 0.03 and 0.07% (marjoram, thyme and oregano), 0.015 + 0.015% (mixture of oregano and thyme), 0.005 + 0.005% (mixture of thyme and BHA), 0.005 + 0.005% (mixture of oregano and BHA), and 0.01% of BHA. Afterwards, the samples were mixed and alcohol was evaporated in a rotary evaporator at 40 °C. The oil samples without extracts added were used as controls. All the prepared oils samples were subjected to DSC oxidation measurements.

### DSC analysis

DSC measurements were conducted with a DSC 820 from Mettler Toledo (Schwerzenbach, Switzerland) with air flow of 60 mL min^−1^. The oils samples with and without the addition of herbal plant extracts and BHA (4.5 ± 0.5 mg) were placed into aluminium pans, closed with lids with a hole drilled in the centre to allow the samples to be in contact with the air stream. The aluminium reference pan as identical as possible to the oil sample pan was left empty. The oil sample and reference pans were heated at the rates of 4.0, 7.5, 10.0, 12.5, and 15.0 °C min^−1^. For each experiment and each programmed heating rates (*β*, °C min^−1^) at least triplicate determinations were carried out. From the resulting oxidation exotherms, the onset oxidation temperatures (*t*_ON, °C_) were determined as the intersection of the extrapolated baseline and the tangent line (leading edge). The *t*_ON_ experimental values were recalculated on absolute onset temperatures (*T*_ON_, K) and it was found that for the samples studied there is a linear correlation (*r*^2^ > 0.938) of the type:

1 $$ { \log }\beta \, = \,a \cdot T_{\text{ON}}^{ - 1} \, + \,b, $$

where *β* is the heating rate (K min^−1^), and *a* and *b* are adjustable coefficients [the slope coefficient from Eq. (1) for each oil samples studied]. The obtained data were used to calculate the apparent activation energy by the Ozawa–Flynn–Wall method from the following equation:

2 $$ E_{\text{a}} \, = \, - \, 2. 1 9\cdot R \cdot \left( {{\text{dlog}}\beta /{\text{d}}T_{\text{ON}}^{ - 1} } \right), $$

where *R* is the universal gas constant (8.314 J mol^−1^ K^−1^). Using the Arrhenius equation [*k* = *Z*·exp·(− *E*_a_ *R*^−1^·*T*^−1^)], the values of rate constants at 160 °C were calculated. Then, they were recalculated into induction time (*τ*) at this temperature. The detailed procedures for kinetic characterisation were reported elsewhere (Thurgood et al. 2007; Kozlowska et al. 2013).

### Statistical analysis

The analyses were conducted in triplicate and the results presented are the average of the values obtained. The multiple range least significant difference test (Duncan multiple range test), with significance level at *P* < 0.05, was applied to the results to test the significant difference. The Statgraphics plus 4.0 package (Statistical Graphics Corp., USA) was used for analysis.

## Results and discussion

### Fatty acid composition

Vegetable oils are rich in unsaturated fatty acids, especially monounsaturated and polyunsaturated fatty acids. They can make oils susceptible to oxidation (Mannekote and Kailas 2012; Sarkar et al. 2015). It is known that the presence of two double bonds in the fatty acids structure may cause 10–40 times faster oxidation than the in presence of one double bond (Szterk et al. 2010). Therefore, the fatty acid composition of sunflower and soybean oils, expressed as saturated, monounsaturated, and polyunsaturated, is summarized in Table 1. The percentage content of saturated fatty acids (SFA) in sunflower oil amounted to 10.7%, while the value obtained for soybean oil was slightly higher (15.5%). It can also be noted that the content of monounsaturated (MUFA) and polyunsaturated (PUFA) fatty acids in soybean oil was slightly lower (25.9 and 58.6%, respectively) in comparison with their content in sunflower oil (28.0 and 61.3%, respectively).

**Table 1:** Parameters of sunflower (SFO) and soybean (SBO) oil samples

Parameter	SFO	SBO
Acid value (AV/mg KOH g^−1^)	0.07 ± 0.01^a^	0.35 ± 0.01^b^
Peroxide value (PV/mmol O_2_ kg^−1^)	1.98 ± 0.12^a^	2.50 ± 0.09^b^
Fatty acids/%		
Myristic (C14:0)	0.1 ± 0.0^a^	0.1 ± 0.0^a^
Palmitic (C16:0)	6.7 ± 0.2^a^	11.1 ± 0.3^b^
Oleopalmitic (C16:1)	0.1 ± 0.0^a^	0.1 ± 0.0^a^
Stearic (C18:0)	3.9 ± 0.2^a^	4.1 ± 0.2^a^
Oleic (C18:1)	27.7 ± 0.4^b^	25.4 ± 0.5^a^
Linoleic (C18:2)	60.2 ± 0.7^b^	53.1 ± 0.6^a^
Linolenic (C18:3)	0.5 ± 0.1^a^	5.0 ± 0.2^b^
Arachidic (C20:0)	0.0 ± 0.0^a^	0.2 ± 0.0^a^
Gadoleic (C20:1)	0.2 ± 0.0^a^	0.4 ± 0.1^b^
Dihomo-γ-linolenic (C20:3)	0.6 ± 0.2^a^	0.5 ± 0.1^a^
∑ SFA/%	10.7 ± 0.4^a^	15.5 ± 0.6^b^
∑ MUFA/%	28.0 ± 0.6^b^	25.9 ± 0.7^a^
∑ PUFA/%	61.3 ± 0.8^b^	58.6 ± 0.7^a^

Data represent mean ± SD (standard deviation) (*n* = 3). Values marked by the different lower case superscript letters within a row denote statistically significant differences (*P* < 0.05)

*SFA* sum of saturated fatty acids, *MUFA* sum of monounsaturated fatty acids, *PUFA* sum of polyunsaturated fatty acids

These values were in agreement with Veronezi et al. (2014) studies. However, Chowdhury et al. (2007) studies showed that the total content of MUFA and PUFA in SBO was similar to our results, but the percentage of monounsaturated and polyunsaturated fatty acids in sunflower oil were different. They reported higher content of MUFA and lower percentage of PUFA in sunflower oil if compared with our studies. Although the content of saturated fatty acids in vegetable oils is desirable to improve their oxidative stability, but taking into account nutritional properties of oils, their presence is undesirable, because they can contribute to increasing the concentration of low-density lipoproteins and the plasmatic cholesterol (Wilke and Clandinin 2005). The major saturated fatty acids that were found in edible oils studied were palmitic (C16:0) and stearic (C18:0). SBO contained higher levels of these fatty acids in comparison with SFO (11.1 and 6.7% of palmitic acid, and 4.1 and 3.9% of stearic acid, respectively). Among MUFA, oleic acid (C18:1) was the main representative and its content ranged from 25.4% in SBO to 27.7% in SFO. However, linoleic (C18:2) and linolenic (C18:3) acids were the dominant fatty acids among PUFA. Linoleic acid amount in SFO was higher than that of 53.1% which was determined in SBO, but linolenic acid was found in smaller amount in SFO. Regarding SBO our results were similar to those reported by other authors (Thurgood et al. 2007; Cordeiro et al. 2013; Sarkar et al. 2015). On contrary, linoleic acid content in SFO studies by Cordeiro et al. (2013) and Asnaashari et al. (2015) was lower than that obtained in the present study. Zambiazi et al. (2007) studying sunflower oils found smaller amounts of oleic acid (15.26 and 16.86%) and higher contents of linoleic acid (71.17 and 70.69%) compared to the results demonstrated in our research. Sunil et al. (2015) also demonstrated the lower level of oleic acid and higher amount of linoleic acid in SFO (23.0 and 66.2% for oleic and linoleic acids, respectively) as compared to the present work. Differences in fatty acid composition of SFO and SBO are mostly determined by plant genotype and environmental conditions, particularly temperature, water supply, and more generally agro-climatic conditions (Castro and Leite 2018; Reena Rani and Sharma 2017; Clemente and Cahoon 2009). The higher level of unsaturated fatty acids in SFO than SBO, especially linoleic acid, makes it more susceptible to oxidation process. Therefore, the addition of antioxidants may be one of the way to inhibit oil autoxidation.

### Oxidative stability of sunflower and soybean oils enriched with herbal plant extracts

**Fig. 1:**
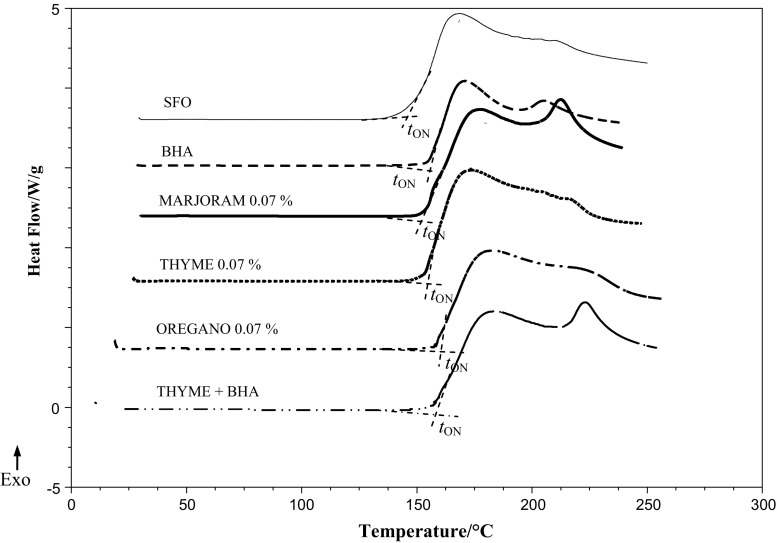
Example of DSC scan of non-isothermal oxidation of sunflower oil (SFO) and SFO samples containing BHA (0.01%), MARJORAM (0.07%), THYME (0.07%), OREGANO (0.07%), and THYME (0.005%) + BHA (0.005%) at heating rate *β* = 4 °C min^−1^

Selected DSC curves of non-isothermal oxidation registered at 4 °C min^−1^ of heating rate for SFO enriched with BHA and herbal plant extracts are shown in Fig. 1. The same profile was also observed for DSC exotherms describing the non-isothermal oxidation of SBO samples. There are usually two peaks present indicating that during oxidation at least two processes take place. The onset point and first maximum peak are connected with initiation and formation of primary auto-oxidation products, and the second maximum peak informs about further oxidation and decomposition of oxidation products (Litwinienko et al. 2000). DSC curves have similar shapes but are shifted towards higher temperatures depending on types and concentrations of herbal plant extracts added.

The start of exothermic reaction temperature values measured as extrapolated onset temperature (*t*_ON_) are summarized in Tables 2 and 3 for SFO and SBO, respectively. *T*_ON_ was reported to be the most suitable parameter for lipid oxidation under non-isothermal oxidation as it is closely associated with the formation of peroxides (Qi et al. 2016). The sample with a higher *t*_ON_ at the same heating rate is more stable than the one for which this parameter is lower. For both vegetable oils studied, an increase of the heating rate resulted in the higher values of *t*_ON_. In the case of SFO samples without the addition of herbal plant extracts they were characterized by lower values compared to SBO samples.

**Table 2:** Extrapolated DSC thermoxidation onset temperatures (*t*_ON_/°C) measured at different heating rates (*β*) for SFO oil samples

Heating rate *β*/ °C min^−1^	SFO	BHA	MARJORAM			THYME		
		0.01%	0.01%	0.03%	0.07%	0.01%	0.03%	0.07%
4.0	145.86 ± 0.47^a^	157.71 ± 0.56^cd^	152.04 ± 0.54^b^	156.17 ± 0.57^c^	156.22 ± 0.57^c^	152.82 ± 0.61^b^	156.68 ± 0.62^c^	156.86 ± 0.84^cd^
7.5	159.37 ± 0.52^a^	164.53 ± 1.00^bc^	159.47 ± 0.97^a^	161.90 ± 1.08^a^	167.97 ± 1.12^c^	162.84 ± 0.87^ab^	166.17 ± 0.89^c^	166.93 ± 0.90^c^
10.0	167.84 ± 0,78^a^	170.01 ± 0.60^ab^	168.74 ± 0.59^a^	170.42 ± 0.91^ab^	172.30 ± 0.92^b^	166.76 ± 1.13^a^	168.02 ± 1.14^a^	168.94 ± 1.15^a^
12.5	171.98 ± 0.56^a^	175.58 ± 0.57^ab^	172.09 ± 0.56^a^	172.42 ± 0.71^a^	174.93 ± 0.72^ab^	172.45 ± 1.44^a^	174.37 ± 1.45^ab^	175.34 ± 1.46^ab^
15.0	176.08 ± 1.17^a^	179.01 ± 1.24^a^	176.10 ± 1.22^a^	177.64 ± 1.23^a^	178.14 ± 1.23^a^	176.52 ± 1.22^a^	177.11 ± 1.48^a^	177.55 ± 1.48^a^

	OREGANO			THYME + BHA	OREGANO + BHA	OREGANO + THYME
	0.01%	0.03%	0.07%	0.005% + 0.005%	0.005% + 0.005%	0.015% + 0.015%
4.0	155.22 ± 0.42^c^	158.44 ± 0.87^d^	159.35 ± 0.69^d^	159.01 ± 0.85^d^	153.65 ± 0.72^b^	155.18 ± 0.70^c^
7.5	163.29 ± 0.58^b^	164.04 ± 0.58^bc^	165.68 ± 0.82^b^	162.62 ± 0.87^ab^	166.13 ± 1.06^bc^	159.72 ± 0.88^a^
10.0	168.01 ± 0.90^a^	169.45 ± 1.39^b^	170.53 ± 1.16^ab^	173.88 ± 1.43^b^	171.68 ± 1.16^b^	167.73 ± 0.85^a^
12.5	172.41 ± 0.85^a^	173.01 ± 0.95^a^	175.48 ± 0.97^ab^	177.49 ± 1.48^bc^	180.03 ± 0.58^c^	172.68 ± 1.10^a^
15.0	176.83 ± 1.00^a^	179.05 ± 1.24^a^	179.41 ± 1.34^ab^	180.22 ± 1.53^ab^	184.39 ± 1.49^b^	176.09 ± 1.32^a^

The results are mean ± standard deviation. Values marked by the different lower case superscript letters (a–d) within a row denote statistically significant differences (*P* < 0.05)

**Table 3:** Extrapolated DSC thermoxidation onset temperatures (*t*_ON_/ °C) measured at different heating rates (*β*) for SBO oil samples

Heating rate *β*/°C min^−1^	SBO	BHA	MARJORAM			THYME		
		0.01%	0.01%	0.03%	0.07%	0.01%	0.03%	0.07%
4.0	157.05 ± 0.58^a^	160.37 ± 0.82^bc^	158.07 ± 0.74^a^	161.37 ± 0.50^c^	162.15 ± 0.57^c^	161.67 ± 0.50^c^	162.18 ± 0.78^c^	162.52 ± 0.78^c^
7.5	164.21 ± 0.67^a^	167.31 ± 0.92^ab^	166.27 ± 0.94^ab^	171.67 ± 1.04^c^	172.06 ± 1.12^c^	169.16 ± 0.74^bc^	169.33 ± 0.74^bc^	171.72 ± 0.75^c^
10.0	170.62 ± 0.79^a^	175.26 ± 0.57^b^	173.06 ± 0.63^a^	174.96 ± 0.64^b^	176.72 ± 0.92^b^	172.78 ± 0.93^a^	173.07 ± 1.08^a^	176.36 ± 0.97^b^
12.5	175.89 ± 0.59^a^	179.53 ± 1.37^b^	178.26 ± 1.26^ab^	179.44 ± 1.22^b^	180.37 ± 0.72^b^	178.76 ± 0.91^b^	179.46 ± 0.94^b^	179.70 ± 0.94^b^
15.0	180.06 ± 1.17^a^	182.35 ± 1.44^ab^	180.36 ± 1.28^a^	182.59 ± 1.29^ab^	183.08 ± 1.23^ab^	181.59 ± 1.21^ab^	181.64 ± 1.26^ab^	182.13 ± 1.26^ab^

	OREGANO			THYME + BHA	OREGANO + BHA	OREGANO + THYME
	0.01%	0.03%	0.07%	0.005% + 0.005%	0.005% + 0.005%	0.015% + 0.015%
4.0	160.98 ± 0.45^bc^	161.25 ± 0.50^c^	161.77 ± 0.53^c^	159.53 ± 0.85^b^	158.75 ± 0.72^ab^	162.75 ± 0.70^c^
7.5	170.17 ± 0.74^c^	170.86 ± 0.79^c^	171.86 ± 0.72^c^	169.04 ± 0.87^bc^	168.01 ± 1.06^b^	171.67 ± 0.88^c^
10.0	174.64 ± 1.09^b^	175.65 ± 1.09^b^	176.54 ± 0.97^b^	172.24 ± 1.43^a^	171.73 ± 1.16^a^	177.84 ± 0.85^b^
12.5	177.05 ± 0.83^ab^	178.07 ± 0.88^ab^	179.58 ± 1.02^b^	174.44 ± 1.48^a^	174.75 ± 0.58^a^	180.23 ± 1.10^b^
15.0	183.03 ± 1.42^ab^	183.09 ± 1.29^ab^	183.13 ± 1.22^ab^	180.11 ± 1.53^a^	180.60 ± 1.49^ab^	185.75 ± 1.32^b^

The results are mean ± standard deviation. Values marked by the different lower case superscript letters within a row denote statistically significant differences (*P* < 0.05)

This observation may be due to the fact that sunflower oil contained higher amount of unsaturated fatty acids, and hence, it was more prone to oxidation process. The addition of herbal plant extracts and synthetic antioxidant to the both oils reduced the oxidation by lengthening *t*_ON_ for antioxidant treated samples. Among herbal plant extracts added to SFO, the best *t*_ON_ values for samples enriched with oregano extracts at concentration of 0.03 and 0.07% were observed. The extrapolated onset temperatures also were increased for SFO samples supplemented with marjoram and thyme extracts, but they were shorter compared to that with the addition of BHA. The significant differences between *t*_ON_ values at the heating rate of 4.0 °C min^−1^ for all sunflower oil samples enriched with herbal plant extracts in comparison with SFO without the addition of herbal plant extracts were observed. However, they were not statistically significant (*P *<0.5) at the same heating rate when SFO samples containing BHA were compared to SFO samples with the addition of herbal plant extracts. However, this observation revealed that the addition of herbal plant extracts to sunflower oil may influence on the improvement of its oxidative stability.

Similar conclusion is possible to make regarding soybean oil samples. In all SBO samples supplemented with herbal plant extracts, the increase of *t*_ON_ was observed compared to the SBO samples without the addition of herbal plant extracts. The highest *t*_ON_ values were shown for SBO enriched with thyme extracts. Moreover, when herbal plant extracts were added to SBO samples at higher concentration (0.07%), the resistance to oxidation was similar to that at concentration of 0.03%. It can also be noticed that the addition of mixture of natural and synthetic antioxidants to the oil samples can cause the prolongation of *t*_ON_ in comparison with oil samples with the addition of BHA and oil samples enriched with each herbal plant extracts separately. The best results were obtained for SFO when it was supplemented with thyme and BHA mixture. In the case, SBO oregano and thyme were the most active.

Herbal plant extracts used in the present study belong to the Lamiaceae family, that is well known for their antioxidant activity due to the content of phenolic compounds. Among phenolics identified by HPLC method in our previously studies, rosmarinic and caffeic acids were the most predominant phenolic compounds (Kozłowska et al. 2014; Kozlowska et al. 2015). Phenolic antioxidants present in herbal plant extracts have good free radical scavenging and chelating properties. When they are incorporated to edible oils help to improve their thermal stability and extend their frying life (Bensmira et al. 2007; Kozlowska and Zawada 2015). Combinations of different herbal plant extracts and synthetic antioxidants may also have synergistic effects in preventing hydroperoxides formation compared to samples containing only herbal plant extracts or only synthetic antioxidant (Rižner Hraš et al. 2000). In a research carried out by Ramalho and Jorge (2008), it was also noticed that rosemary extract added to soybean oil showed a positive effect on its oxidative and thermal stability. However, Abdalla and Roozen (1999) showed that thyme and lemon balm extracts inhibited formation of both primary and secondary oxidation products in sunflower oil and oil-in-water emulsion. Oregano extract was more active in oil than in emulsion.

In the present study, the extrapolated onset temperatures at different heating rates were used to evaluate the thermal-oxidative stability of SFO and SBO enriched with herbal plant extracts. The Ozawa–Flynn–Wall method and the Arrhenius equation were applied for calculation of kinetic parameters of this process. The activation energy (*E*_a_) as well as pre-exponential factor (*Z*) and reaction constant (*k*) can be used for comparison of the oxidative stability of studied pure oils and oils enriched with antioxidants for evaluating of antioxidant activity of added antioxidants. In all cases, the addition of herbal plant extracts and BHA to oils caused an increase in the values of activation energy (Tables 4, 5, respectively). At low concentration of herbal plant extracts (0.01%), they were lower than at higher concentration (0.07%). Activation energy values were 64.86 for SFO and 87.40 kJ mol^−1^ for SBO, respectively. For SFO enriched with herbal plant extracts, *E*_a_ values were in the range of 66.40–101.34 kJ mol^−1^. In the case of SBO containing marjoram, thyme, oregano extracts, and BHA, the range of *E*_a_ values were 88.47–107.30 kJ mol^−1^. The highest values of *E*_a_ were calculated for SBO samples enriched with thyme extract and the mixture of thyme extract and BHA. In regarding to SFO samples, activation energy reached maximal value of 101.34 kJ mol^−1^ when SFO was enriched with oregano extract at concentration of 0.07%. Taking into account the induction time (*τ*) calculated at 160 °C, it can be seen that higher values were reported for oil samples with the addition of herbal plant extracts than for their counterparts without additives. The efficiencies of natural and synthetic antioxidants used for protection of SFO oil samples from thermoxidation increased in the following order: control (SFO) < marjoram 0.01% < thyme 0.01% < oregano + thyme < oregano 0.01% < marjoram 0.03% < oregano + BHA < thyme 0.03% < oregano 0.03% < thyme 0.07% < BHA < marjoram 0.07% < thyme + BHA < oregano 0.07%. For SBO, the rank was in the following sequence: control (SBO) < marjoram 0.01% < oregano + BHA < thyme + BHA < BHA < thyme 0.01% < oregano 0.01% < thyme 0.03% < oregano 0.03% < marjoram 0.03% < oregano 0.07% < marjoram 0.07% < thyme 0.07% < oregano + thyme.

**Table 4:** Regression analysis of DSC data (*a*, *b*, *r*^2^), activation energies (*E*_a_, kJ mol^−1^), pre-exponential factors (*Z*, min^−1^), rate constants (*k*, min^−1^) and induction times (*τ*, min) for SFO samples

Parameters	SFO	BHA	MARJORAM			THYME		
		0.01%	0.01%	0.03%	0.07%	0.01%	0.03%	0.07%
*a*	− 3.562	− 5.153	− 4.407	− 5.051	− 5.117	− 4.725	− 5.446	− 5.347
*b*	9.09	12.53	10.99	12.41	12.49	11.70	13.28	13.04
*r* ^2^	0.997	0.984	0.987	0.973	0.981	0.989	0.971	0.974
*E*_a_	64.86	93.82	80.24	91.97	93.17	86.03	99.16	97.36
log *Z*	7.52	10.85	9.32	10.68	10.76	10.00	11.52	11.28
*k* at 160 °C	0.494	0.346	0.440	0.384	0.337	0.422	0.362	0.348
*τ* at 160 °C	2.024	2.890	2.272	2.604	2.967	2.369	2.762	2.873

	OREGANO			THYME + BHA	OREGANO + BHA	OREGANO + THYME
	0.01%	0.03%	0.07%	0.005% + 0.005%	0.005% + 0.005%	0.015% + 0.015%
*a*	− 5.221	− 5.466	− 5.566	− 4.619	− 3.652	− 4.908
*b*	12.80	13.31	13.50	11.36	9.17	12.12
*r* ^2^	0.991	0.960	0.980	0.938	0.990	0.960
*E*_a_	95.06	99.52	101.34	84.10	66.49	89.36
log *Z*	11.06	11.55	11.73	9.67	7.58	10.40
*k* at 160 °C	0.396	0.354	0.325	0.334	0.364	0.420
*τ* at 160 °C	2.525	2.824	3.076	2.994	2.747	2.380

Parameters *a*, *b,* and *r*^2^ were obtained from plotting log*β* vs. *T*_ON_^−1^ for all samples

**Table 5:** Regression analysis of DSC data (*a*, *b*, *r*^*2*^), activation energies (*E*_a_, kJ mol^−1^), pre-exponential factors (*Z*, min^−1^), rate constants (*k*, min^−1^), and induction times (*τ*, min) for SBO samples

Parameters	SBO	BHA	MARJORAM			THYME		
		0.01%	0.01%	0.03%	0.07%	0.01%	0.03%	0.07%
*a*	− 4.800	− 4.941	− 4.859	− 5.455	− 5.475	− 5.641	− 5.677	− 5.811
*b*	11.79	12.02	11.88	13.15	13.17	13.59	13.67	13.93
*r* ^2^	0.985	0.989	0.995	0.990	0.996	0.981	0.978	0.997
*E*_a_	87.40	89.96	88.47	99.32	99.69	102.71	103.36	105.80
log *Z*	10.08	10.31	10.17	11.38	11.40	11.82	11.89	12.14
*k* at 160 °C	0.348	0.286	0.318	0.255	0.239	0.271	0.264	0.240
*τ* at 160 °C	2.873	3.496	3.144	3.921	4.184	3.690	3.787	4.166

	OREGANO			THYME + BHA	OREGANO + BHA	OREGANO + THYME
	0.01%	0.03%	0.07%	0.005% + 0.005%	0.005% + 0.005%	0.015% + 0.015%
*a*	− 5.406	− 5.411	− 5.437	− 5.893	− 5.408	− 5.140
*b*	13.06	13.05	13.09	14.21	13.13	12.40
*r* ^2^	0.982	0.990	0.994	0.972	0.979	0.992
*E*_a_	98.43	98.52	99.00	107.30	98.47	93.59
log *Z*	11.30	11.29	11.33	12.41	11.37	10.66
*k* at 160 °C	0.270	0.256	0.246	0.295	0.313	0.236
*τ* at 160 °C	3.703	3.906	4.065	3.390	3.194	4.237

Parameters *a, b,* and *r*^2^ were obtained from plotting log*β* vs. *T*_ON_^−1^ for all samples

Kowalski (1993) also evaluated oxidative stability of edible oils after addition of various antioxidants using pressure DSC. Jaswir et al. (2004) showed that addition of pegaga leaves and limau purut to the oil reduced the oxidation by longer *t*_ON_ of antioxidant-treated samples. Suja et al. (2004) demonstrated that sesame cake extract could be used as a substitute for synthetic antioxidant to protect soybean, sunflower, and safflower oils. However, Litwinienko et al. (1997, 1999) evaluated kinetic parameters of linoleic acid thermoxidation in the presence of the phenolic compounds by DSC. The inhibitory effect varies with concentration of compounds added and the range 5–12 mmol mol^−1^ of linoleic acid gave the best results. In general, results presented in this study showed that is possible to improve the oxidative stability of SBO and SFO oils by the addition of herbal plant extracts rich in phenolic compounds.

## Conclusions

With the use of DSC method, the assessment of oxidative stability of sunflower and soybean oils, and also efficiencies of used herbal plant extracts were performed. SFO containing a slightly higher level of unsaturated fatty acids compared to SBO appeared to be less stable. *T*_ON_ values were higher for soybean oil samples without the addition of herbal plant extracts and also when they were enriched with marjoram, thyme, and oregano extracts in comparison with sunflower oil samples. The calculated activation energy values for the oxidation reaction of tested oil samples showed that the effectiveness of used herbal plant extracts varied with the type of herbal plant extract added to oil samples and with their concentration. Oregano extract at concentration of 0.07% was effective for protection of SFO against oxidation and thyme extract at concentration of 0.07% improved the oxidative stability of SBO. Therefore, these herbal plant extracts can be recommended as a potent source of natural antioxidants replacing synthetic antioxidants for protection of edible oils against oxidation.
